# Reliability-Based Evaluation of the Suitability of Polymers for Additive Manufacturing Intended for Extreme Operating Conditions

**DOI:** 10.3390/polym12102327

**Published:** 2020-10-12

**Authors:** Alvaro Rodríguez-Prieto, Ernesto Primera, Manuel Callejas, Ana M. Camacho

**Affiliations:** 1 Department of Manufacturing Engineering, Universidad Nacional de Educación a Distancia (UNED), 28040 Madrid, Spain; amcamacho@ind.uned.es; 2 Department of Industrial Inspection and Technical Assistance, SGS TECNOS, 28042 Madrid, Spain; manuel.callejas@sgs.com; 3 Department of Applied Statistics, University of Delaware, 531 South College Avenue, Newark, DE 19716, USA; eprimera@udel.edu; 4 Machinery and Reliability Institute (MRI), 2149 Adair Ct., Mobile, AL 36695, USA

**Keywords:** polymer selection, design-for-reliability, durability, additive manufacturing, extreme operation

## Abstract

A reliability engineering program must be implemented from the conceptual phase of the physical asset to define the performance requirements of the components and equipment. Thus, in this work, the aim is to find the most optimal solution to manufacture polymer-based parts for the nuclear power industry using additive manufacturing routes. This case study application has been selected because polymers processed by additive manufacturing (AM) can be well suited for nuclear applications. The methodology includes—firstly—an analysis of the suitability of materials based on high-temperature resistance, thermal aging and irradiation tolerance, considering operation conditions. Secondly, an analysis of materials’ processability considering their associated AM routes is performed based on thermal analysis and evaluation of physical properties of materials. A final assessment integrating the in-service suitability and AM processability is performed using a reliability approach, solving different emerging objective conflicts through defined constraints and selection criteria. According to the integrated in-service performance evaluation: Polypropylene-ethylene polyallomer (PPP), Epoxy (EP), Phenolics (Ph), Polyurethane (PU) and Acrylonitrile butadiene rubber (NBR) are the best options for mild operation conditions and EP, Ph and PU, considering high temperature along with radiation exposure. Considering AM techniques: EP and Ph can be manufactured using VAT photopolymerization-stereolithography (VP-SLA) with a good expected processability being these materials valid for high temperature environments. Consequently, this research work analyzes the viability, processability and in-service behavior of parts.

## 1. Introduction

The contemporary engineering design process takes into account the full life cycle of the component being designed. This includes its functions, manufacturability and economic and environmental factors in the product’s life cycle analysis [1]. Engineering design should be based not only on the requirements on safety, but also on environmental aspects of product manufacture, use and disposal [2,3]. Over the past decade, within manufacturing industries there has been an increasing interest in research techniques that allow the performance of processes with high efficiency under sustainable environments [4]. Additionally, in recent years, customers have been paying more and more attention to the green level of a product and incorporating green factors into their choice of living products [5]. In order to ensure sustainable production, the entire process chain must be taken into consideration [6].

Additive Manufacturing (AM) technologies can sustainably contribute to the Fourth Industrial Revolution, providing advantages versus traditional manufacturing processes [7]. AM is becoming a future-oriented manufacturing process because it can produce customized products, allowing high levels of complexity with high precision, resolution and repeatability, reducing material wastes and addressing components’ obsolescence problems, for example, in the nuclear industry. Obsolete parts are particularly well-suited for this new technology as they and their designs are virtually difficult to obtain due to a lack of design information such as component drawings or bill of materials [8,9]. Moreover, the main AM advantage is that parts with more complex geometry can be manufactured without tools [10,11,12]. Thus, AM has drawn tremendous attention from both academia and industry with its potential applications in numerous fields [13].

In addition, another advantage is that at-home 3D printing capitalizes on the elimination of product transport [14]. AM technology eliminates the need of having large spare warehouses, but it requires an additional reliability evaluation to update the failure rate and inspection intervals of the spare components [15]. AM exemplifies a decentralized, on demand, localized and customizable manufacturing model, and its associated sustainability has been demonstrated recently with initiatives such as the home manufacturing of masks, filters and protective visors. Thus, AM may imply major changes for the economy, society and industry of the future [16]. The AM technology proposes major challenges to business models because it is a new approach to traditional business models [17].

Nowadays, polymer materials hold an important role in industry, thanks to their unique properties, such as a wide range of operating temperatures, high thermal/electrical insulation, corrosion-and light-resistance and adequate balance between different mechanical properties such as ductility and mechanical strength [18,19]. Using high-performance polymers for AM is pointless without proper selection of the appropriate AM technique [20]. In addition, the state of the art technique is often insufficient to help us understand the reliability of materials and manufactured parts, and consequently of their associated AM processes [21].

In fact, the lack of AM specific mechanical standards creates challenges for stakeholders to provide intercomparisons between machines, materials and models that predict final part properties to generate valid designs. Focusing on the type of AM technology, 50% of newly AM standards will be related to powder bed fusion (PBF), while 33% will be related to extrusion-based additive manufacturing (EBAM) and the 17% to direct energy deposition (DED) processes [22]. Therefore, the methodology presented in this work is centered in PBF and EBAM technologies. Thus, selective laser sintering (SLS) is chosen from PBF technologies and fused deposition modeling (FDM) is selected from EBAM technologies. DED technologies are discarded since they are predominantly used for metal powders. However, on the other hand, in the study, the VAT photopolymerization (VP)-stereolithography (SLA) technique is included because it is an adequate technique to process thermosets. In addition, SLS can be used for thermoset and elastomer AM [23,24]. VP (including SLA) and EBAM (including FDM) are very suitable routes to additively manufacture with polymers [25]. Due to this reason, thermosets and elastomers are included in the study along with the typical additively processable thermoplastics.

The process of characterizing the suitability of a material and its associated manufacturing technique may involve a bulk of tests that requires a substantial amount of time and the investment of vast quantities of resources [26,27].

The selection problem of the material-AM process binomial can be addressed, not only by performing trial-error testing, but also by performing recommendable previous analysis of suitability [9].

Thus, in this work, the aim is to find the most optimal solution to manufacture polymer-based parts for nuclear power industry using additive manufacturing routes. This case study application has been selected because polymers processed by AM can be well suited for nuclear applications.

The selection problem considers different aspects such as the fitness for service of each candidate material and the match with the considered AM technologies (FDM, SLS, and VP-SLA). To this end, a stringency level methodology integrating multiobjective optimization concepts is used to obtain the best candidates considering an optimal additive manufacturing processing and in-service behavior including reliability calculations.

Thus, a complex analysis is carried out, including an analysis of suitability of materials along with AM route possibilities, considering operation conditions. Some other aspects of materials’ life-cycle impact on the environment, such as material recyclability, are also considered in the study.

Therefore, this research work analyzes the viability, processability and in-service behavior of parts. This novel approach addresses a necessity not covered by most of the multicriteria studies where reliability and quality assurance [28] are not incorporated as key aspects in materials selection in product design.

## 2. Methodology

Finding the optimal solution that combines an adequate in-service behavior along with a cost-efficient result is very complex and requires a multiperspective analysis; since, for example, a large in-operation durability is required together with a short degradation time when the material ends its life cycle. Thus, different constraints (CS) to solve the conflicts between goal functions and selection criteria (SC) are established. Thermoplastics, thermosets and elastomers to be processed using AM are evaluated according to their in-service performance.

**Figure 1:**
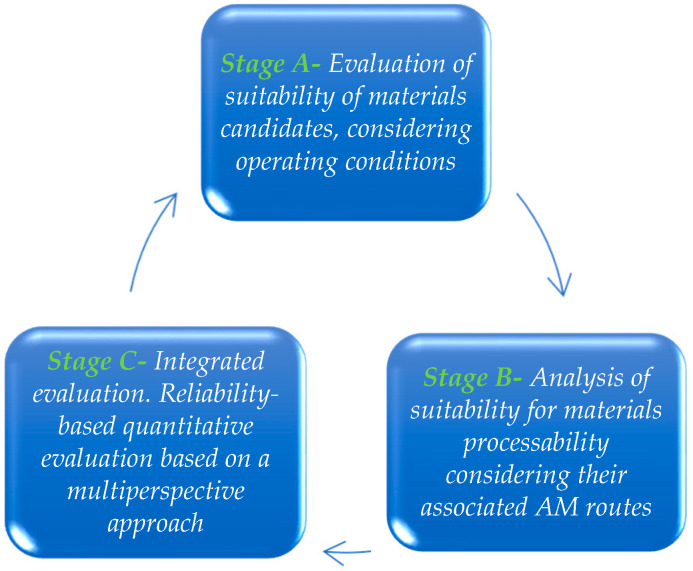
Methodology of analysis.

In addition, it is important to consider human factors in the material life cycle (e.g., obsolescence among others). Cox et al. [29] found that product lifetime depends as much on human factors as it does on functional product durability [30]. AM techniques are well suited to the nuclear industry’s requirements for low volume production, wide variety and highly critical plant components. Thus, obsolete parts are particularly well-suited for this new technology as they and their designs are virtually difficult to obtain due to a lack of design information such as component drawings or bill of materials [8]. Figure 1 provides the methodology of analysis developed in this work.

Prior to data collection, several technological requirements have been selected according to their ability to describe in-service performance related to environmental conditions [24] and the suitability for additive manufacturing processability. Thus, in-service performance parameters (and calculations performed based on them) for further evaluation (Stage A) are divided into three features groups:

- High-temperature resistance (HTR): maximum continuous service temperature (*T_max_*) and the remelting possibility of the polymer candidate.
- Thermal aging tolerance (TAT): based on activation energies specified by EPRI TR 1009748 in this work, useful life at a previously defined upper limit of operating window (*t_failure_*) and remaining useful life (*RUL_Tmax_*) at *T_max_* are calculated.
- Irradiation (gamma) aging resistance (IAT): radiation tolerance threshold (*RTT*) until a decrease of a 50% in ultimate tensile strength (*UTS*) and maximum elongation (*EL*).

On the other hand, material processability (Stage B) analysis is centered on thermal conductivity and thermal diffusivity that allow us to calculate interesting parameters such as the maximum allowable supplied heat (*H_s,max_*) and the maximum linear heat dissipation (*D_L,max_*).

**Figure 2:**
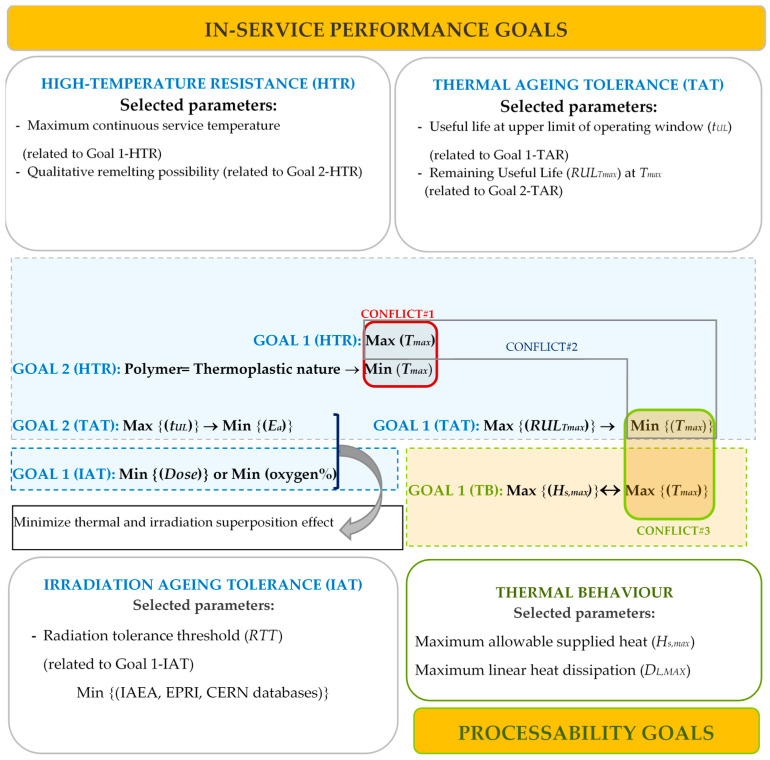
Multiobjective perspective to evaluate goals and conflicts between in-service performance and processability. Abbreviation in Figure: *T_max_*: Maximum continuous service temperature; *t_UL_*: Useful life at upper limit of operating window; *RUL_Tmax_*: Remaining Useful Life at *T_max_*; *Ri*: Radiation tolerance threshold; *E_a_*: Activation energy; *H_s,max_*: Maximum allowable supplied heat; *D_L,MAX_*_:_ Maximum linear heat dissipation; *a*: Thermal diffusivity.

Analysis of in-service related parameters (Stage C) requires a multiperspective approach (Figure 2) since the fulfillment of objective of several functions involves several conflicts. For example, a greater maximum continuous service temperature (*T_max_*) is obviously desirable (increasing high temperature resistance and processing temperature), but the maximizing of this parameter demand, in some cases, a decrease of the remaining useful life at *T_max_* (*RUL_Tmax_*), and implies the preference to use of thermosets implying no possibility of remelting (and, therefore, recyclability).

AM is well-suited to manufacture complex forms, and suitable when design data are not available. Therefore, AM can fit very well for the manufacturing of components intended for the nuclear sector. Most typical components and polymers [9] used in nuclear power plants are:

- Thermoplastics (Polyvinyl Chloride-PVC, Polyamide-PA, Polycarbonate-PC, Polyethylene-PE, Polypropylene-ethylene polyallomer-PPP) for mechanical and electromechanical components.
- Thermosets for sealants, lining in tanks and printed circuit boards (PCB): Epoxy-EP, Phenolics-Ph, Polyester-PL, Polyester (glass filled)-PL-GF and Polyurethane-PU.
- Elastomers for O-Ring manufacturing: Acrylonitrile butadiene rubber-NBR, Fluorocarbon elastomer-FKM, Silicone rubber-SI.

Reviewing the suitable AM techniques, Table 1 exhibits the materials along with their potential application components in the nuclear industry, as well as the possible additive manufacturing routes to process them.

### 2.1. Stage A—Evaluation of Materials Candidates, Considering Operating Conditions

A reliability engineering program must be implemented from the conceptual phase of the physical asset to define the reliability requirements of the systems and equipment and the selection of suppliers that can deliver high-performance equipment in reliability. Therefore, predictive analyses based on more extended and recognized models and databases are performed.

Thermal aging of polymers considering operating time can be explained using the Arrhenius model. Thus, the common form to equate periods of time at two different temperatures is (Equation (1)):

(1) $$t_{UL}^{\prime }=t_{UL}\cdot e^{-\left[\frac{Ea}{k}\left(\left(\frac{1}{T_{n}}\right)\;-\;\left(\frac{1}{T_{a}}\right)\right)\right]}$$

where:

*t_UL_* = Lifetime by design (hours) at normal plant temperature
*t’_UL_* = Useful life (hours) at an increased temperature
*T_n_* = Normal plant temperature (K)
*T_a_* = Augmented temperature (K)
*E_a_* = Activation energy (eV)
*k* = Boltzmann’s constant = 0.8617 × 10^4^ eV/K

The behavior of polymers under irradiation is strongly influenced by the atmosphere in which they are irradiated, particularly in the presence of oxygen. When polymers are irradiated in oxygen-containing atmospheres, the irradiation dose required to reach a particular level of degradation usually changes with dose rate [40]. However, many studies show a synergy between heat and radiation most of the failures are caused by thermal aging rather than radiation aging [41]. Radiation resistance is characterized by the half value dose of significant mechanical properties, for instance tensile strength [42]. This is the absorbed dose that reduces a property by 50% [43]. Thus, several studies have determined radiation thresholds until no severe degradation effects are observed [44,45,46].

**Figure 3:**
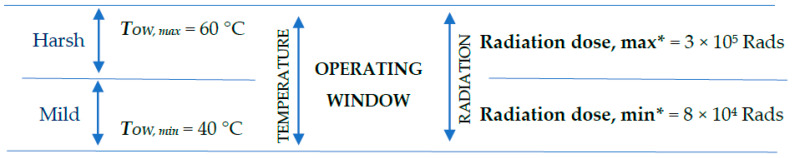
Temperature operating window in mild environment plus radiation (considering normal conditions). Note (*): Main pumps room usual radiation.

Well-defined temperature and radiation operating windows are essential to stablish an analysis framework (Figure 3). Thus, defined normal conditions allow us to consider different scenarios depending the parameters variability inside the constructed range. Additionally, extreme conditions (out of operating windows) can be taken into account, comparatively, with respect to normal operating parameters (considering mild or harsh environment).

Table 2 shows energy activation (*E_a_*) according to EPRI TR 1009748 [47] for thermal aging and maximum continuous service temperature (*T_max_*) [48], along with the radiation thresholds according to EPRI NP 2921 [44], IAEA 1551 [45] and CERN 72-07 [46].

The time to failure (*t_failure_*) is obtained by solving the Equation (2)

(2) $$t_{failure}=\lim_{T_{a\to T_{OW,max}}}t_{UL}\cdot e^{-\left[\frac{E_{a}}{k}\left(\left(\frac{1}{T_{n}}\right)-\left(\frac{1}{T_{a}}\right)\right)\right]}$$

Additionally, Equation (3) is used to calculate *RUL_Tmax_*.

(3) $$RUL_{Tmax}=\lim_{T_{a\to T_{max}}}t_{UL}\cdot e^{-\left[\frac{E_{a}}{k}\left(\left(\frac{1}{T_{n}}\right)-\left(\frac{1}{T_{a}}\right)\right)\right]}$$

Table 3 presents *t_failure_* at *T_OW,max_* and *RUL_Tmax_*.

Using a stringency level methodology [49], the maximum level of stringency is assigned to the maximum value of the distribution (to evaluate HTR and TAT). The stringency level (SL) is therefore calculated according to Equation (4):

*SL* = 5.00 for *max* {*L_s_* (PVC, PA, PC, PE, PPP, EP, Ph, PL, PL-GF, PU, FKM, SI, NBR)} (4)

**Figure 4:**
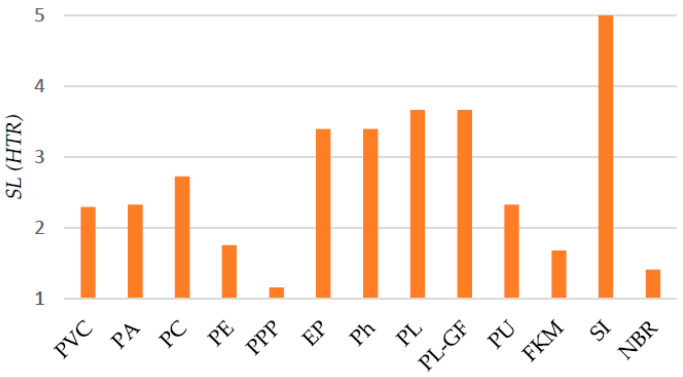
High temperature resistance in terms of stringency level values.

SL of the rest of HRT and TAT requirements are calculated as follows (Equation (5)):

(5) $$SL={\frac{L_{s}}{L_{s}}}_{max}SL_{max}$$

where *L_s_* is the analyzed requirement value, *L_s,max_* is the maximum value of the distribution and *SL_max_* is the maximum value in the scale of stringency levels (between 1 and 5). Thus, Figure 4 exhibits the SL (HTR).

As Figure 2 indicated, conflict#1 and conflict#2 arise when materials should be selected according to the maximum temperature in service (*T_max_*); to maximize the high-temperature resistance (HTR), *T_max_* must be maximized. Nevertheless, this differs to the objective of selecting thermoplastic polymers nature due to their better recyclability. In addition, to maximize a remaining useful life (*RUL_Tmax_*) in abnormal conditions (out of normal operating window; at *T_max_*), *T_max_* should not be a higher value. Therefore, a constraint (CS#1) is now defined to find a moderate balance:

**Constraint (CS)#1**: 2 < *SL*(HTR) ≤ 3.5

Therefore, according to the results shown in Figure 4 and the Constraint #1, the following polymers meet the criteria: PVC (*SL* = 2.30), PA (*SL* = 2.33), PC (*SL* = 2.72), EP (*SL* = 3.40), Ph (*SL* = 3.40) and PU (*SL* = 2.33).

For the TAT requirements, a relative weight of 90% for the normal operating condition versus abnormal operation is considered (in case of accident, i.e., out of normal operating window as indicated in Figure 3), Equation (6) shows the weighted *SL*:

(6) $$SL\left(TAT\right)=0.90\cdot SL\left(t_{failure}atT_{OW,max}\right)+0.10\cdot SL\;\left(RUL_{Tmax}\right)$$

**Figure 5:**
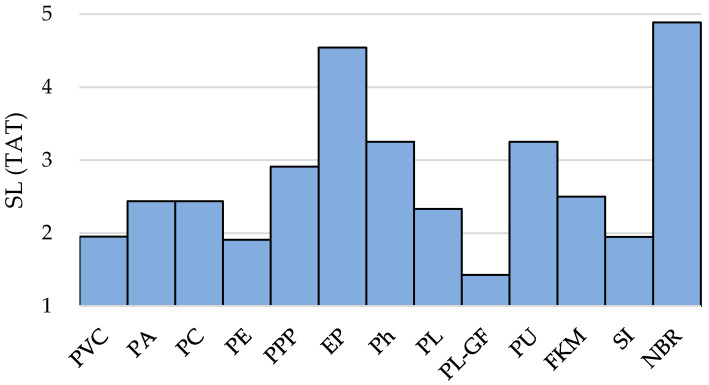
Thermal aging tolerance (TAT) in terms of stringency level values.

Thus, Figure 5 exhibits the stringency level of thermal aging tolerance—*SL* (*TAT*)—for the analyzed materials set.

According to the results shown in Figure 5 along with a goal function that consists in maximizing *t_failure_* and *RUL_Tmax_*, the materials with highest values (above *SL*(*TAT*) ≥ 3.00) are: PPP (*SL* = 3.02), EP (*SL* = 4.30), Ph (*SL* = 3.08), PU (*SL* = 3.08), NBR (*SL* = 4.77). 

Equation (7) provides the calculation of *RTT_i_* based on the more restrictive (minimum) value provide by IAEA, EPRI and CERN databases.

(7) $$RTT_{i=min\left\{R_{max,IAEA\;1551},\;\;R_{max,EPRI\;NP\;2921},\;R_{max,CERN\;72\;-\;07}\right\}}$$

Irradiation performance is calculated applying a stringency level calculation. It assigns the maximum level of stringency to the maximum value of the distribution, according to the following equation:

The calculation to evaluate the radiation resistance is developed according to the Equations (8)–(12) using radiation threshold (*R_OW,max_*) and the radiation tolerance threshold for the polymer *i* according to IAEA, EPRI and CERN databases (*RTT_i_*), that defines the limit to avoid pernicious effects on the mechanical properties of the polymers:

*SL* = 1.00 (*RTT_i_* < *R_OW,max_*) (8)

*SL* = 2.00 (*R_OW,max_* ≤ *RTT_i_* < 1.75 *R_OW,max_*) (9)

*SL* = 3.00 (1.75 *R_OW,max_* ≤ *RTT_i_* < 2.75 *R_OW,max_*) (10)

*SL* = 4.00 (2.75 *R_OW,max_* ≤ *RTT_i_* < 3.75 *R_OW,max_*) (11)

*SL* = 5.00 (*RTT_i_* ≥ 3.75 *R_OW,max_*) (12)

Therefore, a safety factor (*SF*) equal to 3.75 is chosen to assign maximum stringency value.

**Figure 6:**
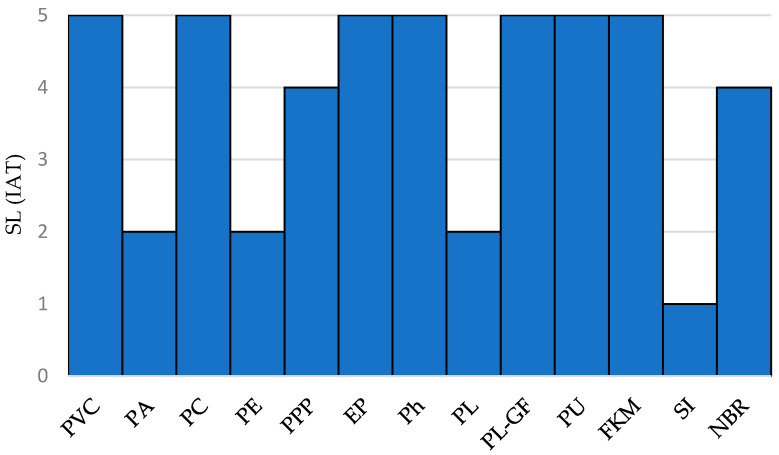
Irradiation aging tolerance based on the stringency level approach.

Figure 6 shows the irradiation aging tolerance based on the stringency level approach.

IAT evaluation is shown in Figure 6, in terms of stringency levels using Equations (8)–(12). Thus, selecting the polymers that exhibit *SL*(IAT) ≥ 3, the following list is obtained: PVC (*SL* = 5.00), PC (*SL* = 5.00), PPP (*SL* = 4.00), EP (*SL* = 5.00), Ph (*SL* = 5.00), PL-GF (*SL* = 5.00), PU (*SL* = 5.00), FKM (*SL* = 5.00), NBR (*SL* = 4.00).

### 2.2. Stage B—Analysis of Materials Processability Considering Their Associated AM Routes

As mentioned previously, FDM, SLS and VP-SLA have been selected as the potential AM routes to process the materials when obsolescence problems and an intricate manufacturing geometry are challenges to face.

FDM uses a heated nozzle to convert the thermoplastic filament into a semi-molten form, which is extruded to build the structure through layer-by-layer deposition [50]. Compared to other AM methods, as the FDM method offers many advantages including cost effectiveness, and now, many researchers are moving towards FDM to study this process thoroughly [51].

With SLS, a powder bed fusion AM process, three-dimensional (3D) objects can be fabricated by adding powdered materials layer by layer according to computer-aided design (CAD) models [52]. The main advantage of SLS is that it can process an extensive scope of materials including polymers, metals, ceramics and composites. In fact, SLS of plastics shows an excellent capability to manufacture serial components.

VP-SLA is a very high suitable technique for thermoset and elastomers additive manufacturing, whereas PBF or EB are not suitable. VP-SLA is one of the most interesting technologies among the wide AM family, because it allows one to reach the highest levels of accuracy combining the advantages of starting from a liquid base material with the vector scanning driven modality that the laser adopts to cure it [53].

For example, the photopolymer resins used during this process are also known as thermosetting plastics or thermosets, which strengthen during post-curing and keep their shape, even after reheating. This is in contrast to thermoplastics, which can be re-melted after being formed into a part.

Some recent AM studies have been centered on thermal properties, calculating the thermal profiles for an arbitrary rastering sequence, squarely focusing on part scale depending on several normalized parameters that depend on the time and length scales of the rastering process: normalized power, normalized thermal diffusivity and normalized beam width. Normalized profiles remove a prefactor, resulting on profiles that depend on two parameters, the normalized thermal diffusivity and the normalized beam spread [54]. Therefore, in this work, thermal diffusivity is considered as the most relevant feature to calculate thermal processability.

The differential equation of heat conduction through an isotropic material [55] considering the propagation in a linear dimension, is shown in Equation (13).

(13) $$\nabla^{2}kT=k\frac{\partial^{2}T}{\partial x^{2}}=\rho c\frac{\partial T}{\partial t},\;x\in \left[0,\;\mathrm{thickness}\right]\;\mathrm{and}\;t>0$$

where ρ is the material density and *c* is the heat capacity of material.

Thus, an important processability parameter is the density of polymer, since the mechanical strength typically increases with the molecular weight and degree of branching or side chains. The other important parameter is the specific heat capacity of material (*c_s_*), presented also in Table 4, along with density, according to the information provided by different databases [44,45,46].

Other aspects important in the further processing are related to the rheological behavior of materials. Nevertheless, this methodology intends to fit a basis to make a decision on materials and their associated AM routes. Rheological aspects can be quantified by an experimental or numerical method in a further phase.

Table 4 shows the main processability parameters (density-ρ, thermal conductivity-*k*, thermal diffusivity-*a*, specific heat capacity-*c_s_*, typical layer thickness per AM technique-*t_layer,min_* obtained from different databases [48,56,57,58,59,60,61,62,63,64,65,66,67], including the calculation of penetration depth (*d*).

Subsequently, it is recommendable that a medium density is used to find a balance between mechanical strength and processability. Unfortunately, this also results in an elevation of the melt viscosity and melting point, and the extrusion of high strength thermoplastics requires elevated temperatures that aggravate thermal distortion [56]. Therefore, an adequate balance between resistance and elongation is crucial to improve processability, delaying also an early embrittlement due to a further thermal and radiation in-service degradation. Thus, (SC)#1 is stablished considered a balanced requirement mean ±0.2.

**Selecting criterion (SC)#1:** 1.25 < *ρ* ≤ 1.65

Therefore, PVC, PC, PE, EP, Ph, PL, PL-GF, PU and SI fulfill the selection criterion (SC)#1.

On the other hand, it is recommendable a low specific heat capacity to ease the heat transfer. The following selection criterion is considered in the analysis, considering that the study is comparative and a *c_s_* = 1500 J/kg·°C is a central representative value.

**Selecting criterion (SC)#2:***c_s_* < 1500 J/kg·°C

Thus, the selection order according to SC#2 criterion is: PVC (*C_s_* = 1004 J/kg·°C), FKM (*C_s_* = 1023 J/kg·°C), EP (*C_s_* = 1046 J/kg·°C), PL/PL-GF (*C_s_* = 1130 J/kg·°C), SI (*C_s_* = 1255 J/kg·°C), NBR (*C_s_* = 1443 J/kg·°C) and Ph (*C_s_* = 1463 J/kg·°C).

On the one hand, high thermal conductivity improves processability and, on the other hand, heat transfer and hence reduce the inside temperature and thermal stress of components [57]. Thermal analysis can become a useful tool to enhance our ability to predict the outcome of the fabrication process [54]. In additive manufacturing, parameters such as thermal conductivity and diffusivity and specific heat capacity are essential to estimate the penetration depth (mm) and speed (mm/s), which is important to evaluate the suitability of different additive manufacturing routes. Thus, penetration depth (*d*) can be calculated according to Equation (14):

(14) $$d=k\sqrt{a\cdot t}$$

where *a* is the thermal diffusivity, *t* is the application time of energy-input and *k* is the thermal conductivity.

In addition, considering the maximum service temperature (*T_max_*), the maximum allowable supplied heat (*H_S, MAX_*) can be calculated according the Equation (15).

(15) $$H_{S,\;\;MAX}=\Delta T_{MAX}\cdot c_{s}$$

where *c_s_* is the specific heat capacity, calculated as c/*m* (where *m* is the mass in kg and *c* is the heat capacity in J/°C) and *ΔT_MAX_* is the maximum temperature gap, calculated like the difference between *T_MAX_* (maximum service temperature—until degradation) and usual room temperature (25 °C).

**Figure 7:**
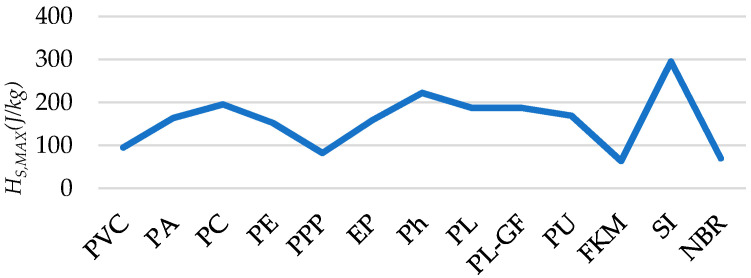
Maximum allowable supplied heat (*H_S,MAX_*).

Figure 7 provides the *H_S,MAX_* (J/kg) for each analyzed polymer.

To find the most suitable (best processability) options, a selection criterion (SC#3) considering the value of *H_S,MAX_* is established:

**Selection criterion (SC)#3:***H_S,MAX_* > 150 J/g

Analogously, a considered central value of 150 J/g are stablished as a minimum threshold.

Thus, the selection order according to SC#3: SI (*H_S,MAX_* = 294.93 J/g), Ph (*H_S,MAX_* = 222.04 J/g), PC (*H_S,MAX_* = 195.31 J/g), PL/PL-GF (*H_S,MAX_* = 187.08 J/g), PU (*H_S,MAX_* = 168.87 J/g), PA (*H_S,MAX_* = 163.39 J/g), EP (*H_S,MAX_* = 158.65 J/g) and PE (*H_S,MAX_* = 152.12 J/g).

**Figure 8:**
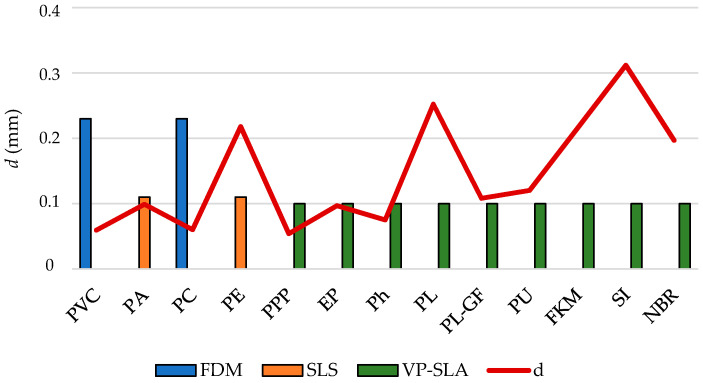
Heat penetration depth (*d*) versus expected layer thickness (*t_layer,min_*) for the considered candidate AM techniques.

One challenge in AM is to control the heat penetration depth since layers are very thin and usually heat input affects more deeply that it would be recommended, moreover considering the discontinuous structure of AM parts [68]. Therefore, to analyze the suitability of each potential AM technique for each polymer, a comparison between heat penetration depth (due to polymer characteristics) and the average expected layer thickness is performed (Figure 8).

Thermoplastics show, in general (excepting PE), better behavior since the penetration depth for a heat input per second is usually less than the average layer thickness. On the other hand, EP and Ph thermosets show similar behavior compared to thermoplastics.

Compatibility between material and their associated AM technique: for FDM, PVC and PC are the best options to control the heat input. For SLS, PA is the unique adequate option. On the other hand, considering the thermoplastic PPP and thermosets and elastomers to be processed using VP-SLA, only PPP, EP and Ph are suitable considering this heat input control criterion.

## 3. Results

In Stage A, performance features of materials have been analyzed, whereas AM processability characteristics have been studied in Stage B obtaining several constraints. In Stage C, an integrated evaluation using a reliability-based approach is carried out using the calculations obtained in stages A and B. Since it is essential to predict reliability of end-products because this is one of their major limitations [69].

### Stage C—Integrated evaluation. Reliability-Based Quantitative Evaluation Based on a Multiperspective Approach

Using the *t_failure_* for operation near upper limit of operating window (60 °C), failure rate *λ_OW,max_* is calculated according to Equation (16).

(16) $$\lambda_{OW,max}=\frac{1}{t_{failure}}$$

If remaining useful life at *T_max_* (*RUL_Tmax_*) is used in the calculation, failure rate at *T_max_* can be calculated as follows (Equation (17)).

(17) $$\lambda_{Tmax}=\frac{1}{RUL_{Tmax}}$$

Table 5 exhibits *λ_OW,max_* and *λ_Tmax_* for each material.

Using a normal distribution, the reliability function *R*(*t*) is calculated according to Equation (18).

(18) $$R\left(t\right)=e^{-\lambda t}$$

On the other hand, probability of failure distribution *POF* (*t*) can be calculated according to Equation (19).

(19) $$POF\;\left(t\right)=1-e^{-\lambda t}=1-R\left(t\right)$$

**Figure 9:**
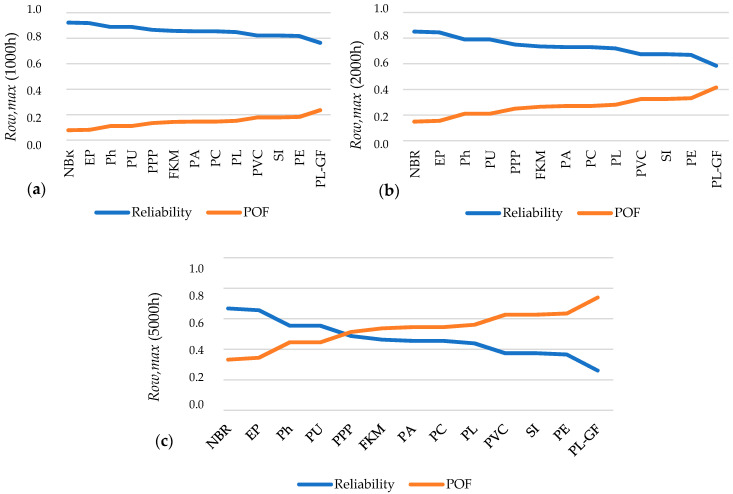
Reliability (*R*) and Probability of Failure (*POF*) for 1000 h (**a**), 2000 h (**b**) and 5000 h (**c**) under the maximum operating window temperature (*T_ow,max_*).

Figure 9 shows the reliability (*R*) and probability of failure (*POF*) for 1000 h (a), 2000 h (b) and 5000 h (c) of operating time.

According to the results shown in Figure 9, the following selection criteria are defined:

**Selection criterion (SC)#4:***R_ow,max_* (2000 h) > 0.75 when T → *T_ow,max_*

**Selection criterion (SC)#5:***R_ow,max_* (5000 h) > 0.50 when T → *T_ow,max_*

Hence, for in-service reliability at *T_ow,max_*, a typical reliability greater than 0.75 for 2000 h and 0.50 for 5000 h are required.

Thus, the selection order according to SC#4 is NBR (*R* = 0.85), EP (*R* = 0.84), Ph (*R* = 0.78), PU (*R* = 0.78) and PPP (*R* = 0.75), and the selection order according to SC#5 is NBR (*R* = 0.67), EP (*R* = 0.65), Ph (*R* = 0.55) and PU (*R* = 0.55).

**Figure 10:**
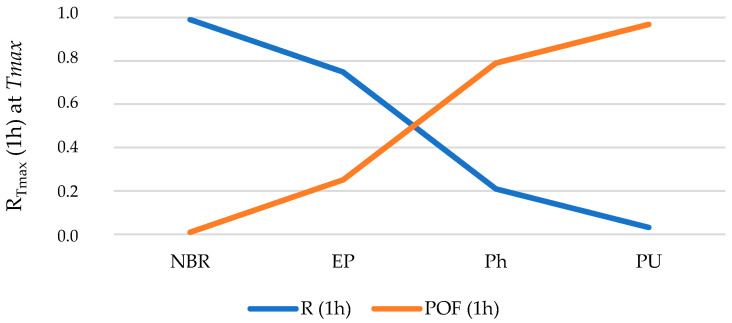
Reliability (*R*) and Probability of Failure (*POF*) after 1 h of operating at (*T_max_*).

If NBR, EP, Ph and PU (that meets SC#5, i.e., 5000 h at *T_ow,max_*) are analyzed considering the reliability at abnormal conditions (i.e., at *T_max_* for each material), using the *λ_Tmax_* presented in Table 5, the results obtained are as follows (Figure 10).

Analogously, a selection criterion is established for operation at *T_max_*:

**Selection criterion (SC)#6: ***R_Tmax_* (1 h) > 0.50 when T → *T_max_*

Analogously, a minimum reliability of 0.50 for 1 h at *T_max_* considering that this is an extreme event.

An analysis of in-service and processability parameters requires a multiobjective approach since the fulfillment of the objective of several functions involves several conflicts. Hence, Table 6 shows a collection of the conflicts (C#) emerged along with the constraints (CS#) defined to solve them. In addition, selection criteria emerged during the course of the methodology are indicated as a note of the Table. These six selection criteria have been selected for being representative of general aspects related to processability and in-service behavior.

Using the selection criteria (SC)#1, 2 and 3, Table 7 provides the materials and AM techniques suitability according to the evaluation of heat input control performed in Stage B (Section 2.2).

Additionally, Table 8 exhibits the selected materials according to their thermal and radiation tolerance (Stage A—Section 2.1), using the selection criteria (SC)#4, 5 and 6.

**Figure 11:**
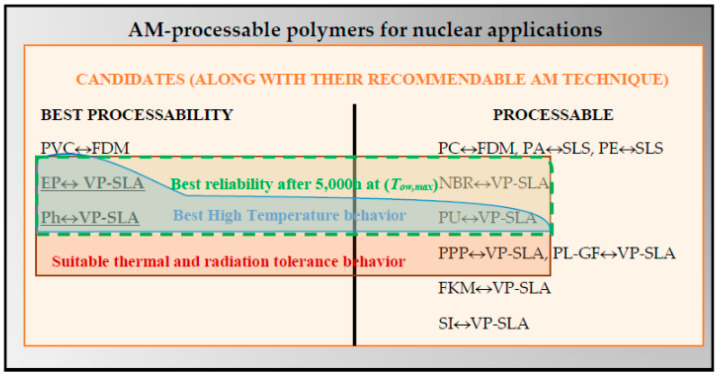
Material selection chart for nuclear applications.

According to the evaluation presented in Table 8 (focusing on in-service performance): PPP, EP, Ph, PU and NBR are the best options for mild operation conditions (medium temperature plus radiation) and EP, Ph and PU, considering high temperature along with radiation exposure. If an AM technique is necessary (evaluation presented in Table 7) to reproduce the intricate geometry, EP and Ph can be manufactured using VP-SLA with a good expected processability as these materials are valid for a high temperature environment. Other possibilities with poorer expected processability include PU processed by VP-SLA, also valid for high temperatures, or PPP or NBR processed by the same AM technique but only valid for medium temperature operation. Figure 11 provides a selection chart showing graphically the alternatives and the valuation, being a valuable contribution for the design-for-reliability (*DfR*) applied to the considered case study.

It is important to highlight that advances in materials are promoting the use and applications of additive manufacturing [70]. In addition, numerous innovative techniques were spotted amongst recent work trying to overcome different challenges with material and manufacturing techniques [71]. However, there are still some limitations, especially pertaining to the lower mechanical properties generally exhibited by AM parts [72]. Subsequently, this selection chart can improve the preliminary process and materials integrated analysis.

The averaged stringency level (considering the suitability of thermal and radiation tolerance behavior) can be calculated as (Equation (20)):

(20) $$\begin{array}{cc} SL_{mean}\left(SC\#\right)= & C_{1}\cdot SL\left(SC\#1\right)+C_{2}\cdot SL\left(SC\#2\right)+C_{3}\cdot SL\left(SC\#3\right)+C_{4}\\ & \cdot SL\left(SC\#4,SC\#5,\;SC\#6\right)] \end{array}$$

where:

SL(SC#1 and SC#3) is calculated using Equations (4) and (5).
SL(SC#2) is calculated using Equations (21) and (22) (as follows):
  *SL* = 5.00 for *min* {*L_s_* (PPP, EP, Ph, PU, NBR)} (21)

The stringency levels of the remaining values are calculated as follows:

(22) $$SL=\frac{L_{s_{Min}}}{L_{s}}SL_{Max}$$

SL (SC#4-SC#5 and SC#6) can be calculated (considering a relative weight of 90% for the normal operating condition versus abnormal operation) using the following expression (Equation (23)):

(23) $$SL\left(R\right)=\left[0.90\cdot R_{OW,max}\left(5000h\right)+0.10\cdot R_{Tmax}\left(1h\right)\right]\cdot SL_{,Max}$$

*C*_1_, *C*_2_, *C*_3_ and *C*_4_ are relative weights that can be modulated by decision maker/designer in each case, but always keeping Σ*C_i_* = 1. In this case study, a balanced criterion has been used, therefore *C*_1_ = *C*_2_ = *C*_3_ = *C*_4_ = 0.25 (considering a 75% of weight for processability and 25% for in-service reliability; since not allowable in service-behavior has been limited during the analysis using different constraints and, in this step, processability is essential).

**Figure 12:**
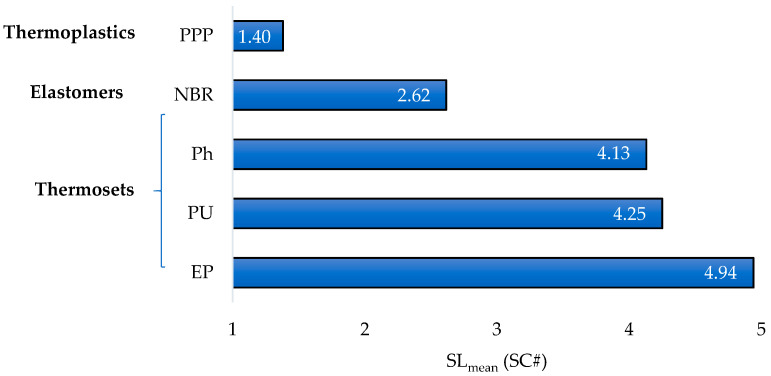
Stringency levels (obtained from selection criteria SC#1 to SC#6).

Figure 12 provides the *SC_mean_* for the materials that meet the constraint (CS)#1. Consequently, the thermosets EP, Ph and PU exhibit the best balance between processability based on AM and in-service behavior in nuclear applications. Although, PPP and NBR show less balanced suitability, they provide a higher reliability at their *T_max_*. Finally, EP and Ph are the best options (according to the selection chart—Figure 11) to be processed using AM techniques (VP-SLA).

## 4. Conclusions

This paper provides a methodology that support designers to make a decision about to manufacture polymer-based parts for nuclear power industry using additive manufacturing routes. A high demanding application such as the manufacturing of nuclear parts has been selected because polymers processed by AM can be well suited for nuclear applications. Thus, a new reliability-based approach using multiperspective concepts has been developed. This methodology eases the complex problem of selecting materials and their associated AM techniques.

Novel performance indicators related to processability and in-service behavior prediction have been integrated in the analysis. These performance indicators along with the definition of several constraints and section criteria have allowed us to solve several conflicts emerged among in-service and processability goals. To quantitatively assess, a stringency level methodology has been defined and integrated in the research methodology.

According to the integrated in-service performance evaluation, PPP, EP, Ph, PU and NBR are the best options for mild operation conditions (medium temperature plus radiation) and EP, Ph and PU, considering high temperature along with radiation exposure.

If an AM technique is necessary to reproduce the intricate geometry, EP and Ph can be manufactured using VP-SLA with a good expected processability being these materials valid for high temperature environment. Other possibilities with poorer expected processability include PU processed by VP-SLA, which is also valid for high temperatures, or PPP or NBR processed by the same AM technique but only valid for medium temperature operation.

The methodology developed in this paper provides a reliability-based analytical tool that allows us to make a decision about the optimal combination of polymer and their associated AM technique along with a prediction of the materials in-service behavior. In future, this methodology and obtained results will be used to improve the materials and AM process selection tasks that are previously performed to the experimental testing.

## Figures and Tables

**Table 1:** Polymers, related applications and AM technique suitability [9,23,24,31,32,33,34,35,36,37,38,39].

Application	Polymer	AM Technique Suitability		
		FDM	SLS	VP-SLA
Mechanical and electromechanical components (fittings and fix pieces)	PVC	X		
	PA		X	
	PC	X		
	PE		X	
	PPP			X
Complex elements of printed circuit boards	EP			X
	Ph			
	PL			X
	PL-GF			
	PU			
Complex diagrams of valves	FKM			
	SI			X
	NBR			

**Abbreviations in Table:** PVC—Polyvinyl Chloride, PA—Polyamide, PC—Polycarbonate, PE—Polyethylene, PPP—Polypropylene-ethylene polyallomer, EP—Epoxy, Ph—Phenolics, PL—Polyester, PL-GF—Polyester glass filled, PU—Polyurethane, FKM—Fluorocarbon elastomer, SI—Silicone, NBR—Acrylonitrile butadiene rubber.

**Table 2:** Evaluation of parameters of degradation (due to thermal and irradiation conditions) [45,46,47,48].

		Thermal Aging and High-Temperature Resistance		Irradiation Aging		
Type	Polymer	*E_a_* (eV)	(*T_max_*) [°C]	*R_max_*_IAEA 1551_ (Rads)	*R_max_*_EPRI NP 2921_ (Rads)	*R_max_*_CERN 72-07_, (Rads)
Thermoplastics	PVC	1.28	119.44	1.0 × 10^7^	1.2 × 10^6^	2.0 × 10^7^
	PA	1.18	121.11	7.0 × 10^5^	7.0 × 10^6^	5.0 × 10^5^
	PC	1.18	141.67	4.0 × 10^6^	7.0 × 10^5^	4.0 × 10^6^
	PE	1.29	91.11	1.0 × 10^7^	3.8 × 10^5^	1.0 × 10^7^
	PPP	1.14	60.00	2.0 × 10^6^	1.0 × 10^6^	2.0 × 10^6^
Thermosets	EP	0.90	176.67	2.0 × 10^9^	2.0 × 10^8^	4.0 × 10^8^
	Ph	1.05	176.67	4.0 × 10^6^	-	2.0 × 10^6^
	PL	1.20	190.56	5.0 × 10^5^	5.0 × 10^5^	4.0 × 10^5^
	PL-GF	1.42	190.56	1.0 × 10^9^	4.0 × 10^8^	1.0 × 10^9^
	PU	1.05	121.11	1.0 × 10^9^	6.0 × 10^7^	1.0 × 10^9^
Elastomers	FKM	1.17	87.22	5.0 × 10^6^	-	-
	SI	1.28	260.00	1.0 × 10^8^	1.0 × 10^5^	1.0 × 10^8^
	NBR	0.88	73.33	2.0 × 10^6^	1.0 × 10^6^	2.0 × 10^7^

**Table 3:** Calculated *t_failure_* and *RUL_Tmax_*.

Polymer	*t_failure_* at *T_OW,max_* (h)	*RUL_Tmax_* (h)
PVC	5082.40	5.95
PA	6348.75	10.86
PC	6348.75	1.94
PE	4970.57	107.07
PPP	6939.63	6939.63
EP	11,836.51	3.48
Ph	8478.01	0.64
PL	6072.45	0.05
PL-GF	3722.01	0
PU	8478.01	0.29
FKM	6491.58	298.82
SI	5082.40	0
NBR	12,375.07	3805.11

Note: Calculation parameters: *t_UL_* = 10 years, *T_n_* = 40 °C (typical operating temperature). *RUL_Tmax_* = remaining useful life (at maximum temperature).

**Table 4:** Analysis of parameters related to materials processability [48,56,57,58,59,60,61,62,63,64,65,66,67].

	Polymer	Density (g/cm^3^)	*K* [W/m·°C]	*a* [mm^2^/s]	*c_s_* [J/kg·°C]	*t_layer,min_* (mm) per AM Technique	*d* (mm), per Second
Thermoplastics	PVC	1.45	0.25	0.18	1004	**FDM:** 0.05–0.4 mm **SLS:** 0.10–0.12 mm	0.06
	PA	1.15	0.24	0.17	1700		0.10
	PC	1.44	0.20	0.09	1674		0.06
	PE	1.38	0.50	0.19	2301		0.22
	PPP	0.91	0.22	0.06	2343		0.05
Thermosets	EP	1.29	0.25	0.15	1046	**VP-SLA:** 0.50–0.15 mm	0.10
	Ph	1.30	0.25	0.09	1464		0.08
	PL	1.50	0.70	0.13	1130		0.25
	PL-GF	1.80	0.30	0.13	1130		0.11
	PU	1.50	0.19	0.40	1757		0.12
Elastomers	FKM	2.00	0.45	0.23	1023	**VP-SLA:** 0.50–0.15 mm	0.22
	SI	1.35	0.75	0.46	1255		0.31
	NBR	1.24	0.42	0.22	1443		0.20

**Table 5:** *λ_OW,max_ andλ_Tmax_* calculation.

Polymer	*λ* *_OW,max_*	*λ* *_Tmax_*
PVC	0.00019	0.16801
PA	0.00015	0.09201
PC	0.00015	0.51546
PE	0.00020	0.00933
PPP	0.00014	0.00014
EP	0.00008	0.28736
Ph	0.00011	1.56250
PL	0.00016	20.0000
PL-GF	0.00026	∞
PU	0.00011	3.44828
FKM	0.00015	0.00335
SI	0.00019	∞
NBR	0.00008	0.00026

**Table 6:** Review of the conflicts solving roadmap.

Goal	Conflict (C)#	Constraint (CS)# to Solve the Conflict# or Minimize the Damage and/or Maximize Processability
HTR-1 Max (*T_max_*)	C#1, C#2, C#3	CS#1 to solve C#1, C#2 and C#3: 2 < *SL(HTR*) ≤ 3.5
TAT-1 Max{(*RUL_Tmax_*)} → Min {(*T_max_*)}		
HTR-2 Polymer = Thermoplastic nature → Min (*T_max_*)		
TB-1 Max {(*H_s,max_*)} ↔ Max {(*T_max_*)}		
TAT-2 Max{(*t_UL_*)} → Min {(*E_a_*)}	-	Minimize thermal and irradiation superposition effect. Selecting polymers with low *Ea*
IAT-1 Min{(*Dose*)} or Min (oxygen%)		

Note: Selection criteria applied for processability = (SC)#1: 1.25 < r < 1.65, (SC)#2: *c_s_* < 1500 J/kg·°C; (SC)#3: *H_S,MAX_* > 150 J/g; Selection criteria applied for in-service reliability = (SC)#4: *R_ow,max_*(2000 h) > 0.75 when T → *T_ow,max_*; (SC)#5: *R_ow,max_* (5000 h) > 0.50 when T → *T_ow,max_*; (SC)#6: *R_Tmax_* (1 h) > 0.50 when T → *T_max_*.

**Table 7:** Materials and techniques according to processability analysis.

Processability and Heat Input Control Evaluation	Materials and Techniques
Best processability options (Selection criteria (SC)#1, 2 and 3)	PVC ↔ FDM, EP ↔ VP-SLA, Ph ↔ VP-SLA.
Other possibilities of processing	PC ↔ FDM, PA, PE ↔ SLS, PPP, PL-GF, PU, FKM, SI, NBR ↔ VP-SLA

**Table 8:** Selected materials according to their thermal and radiation tolerance.

Operation Environment	Selected Materials
Medium temperature + radiation *1	PPP, EP, Ph, PU and NBR.
High Temperature + radiation *2	EP, Ph and PU.

Note *1: not considering HTR criterion; considering thermal aging resistance (TAT). The materials meet SC#4 criterion (2000 h at *T_ow,max_*). Note *2: considering HTR criterion; considering thermal aging resistance (TAT). The materials meet SC#5 (5000 h at *T_ow,max_*) and SC#6 (1 h at *T_max_*).
